# Minimally Invasive Thoracic Corpectomy: Surgical Strategies for Malignancy, Trauma, and Complex Spinal Pathologies

**DOI:** 10.1155/2012/213791

**Published:** 2012-07-24

**Authors:** Rohan R. Lall, Zachary A. Smith, Albert P. Wong, Daniel Miller, Richard G. Fessler

**Affiliations:** ^1^Department of Neurological Surgery, Northwestern University McGaw Medical Center, Chicago, IL 60208, USA; ^2^University of California San Francisco-Fresno Community Hospital of Fresno, San Francisco, CA 94143, USA

## Abstract

The rapid expansion of minimally invasive techniques for corpectomy in the thoracic spine provides promise to redefine treatment options in this region. Techniques have evolved permitting anterior, lateral, posterolateral, and midline posterior corpectomy in a minimally invasive fashion. We review the numerous techniques that have been described, including thoracoscopy, tubular retraction, and various instrumentation techniques. Minimally invasive techniques are compared to their open predecessors from a technical and complication standpoint. Advantages and disadvantages of different approaches are also considered, with an emphasis on surgical strategies and nuance.

## 1. Background

The unique anatomy and structural support in the thoracic spine create challenges for practitioners attempting surgery in the region. Due to the inherent rigidity of the region granted by the rib cage, spondylotic changes are significantly less common than in the cervical and lumbar spine [1]. The most common pathologies in the thoracic spine requiring corpectomy are tumors, trauma, and infection [2–4]. Treating these pathologies can require significant anterior reconstruction, made challenging due to the ribs and other adjacent critical structures including the lungs, pleura, aorta, and mediastinum [5]. Obtaining adequate exposure for corpectomy is critical due to the relative intolerance of the thoracic spinal cord to manipulation and mobilization [1, 3, 6]. Additionally, the numerous comorbidities usually present in these patients often preclude the systemic stress of open surgery [7].

 Minimally invasive techniques in the cervical and lumbar spine have been clearly demonstrated to lower surgical blood loss, pain, improve wound healing, and shorten hospital stay [8–10]. In the thoracic spine, their advent is allowing surgeons to consider treatment for patients who previously would have been relegated to bracing and palliative pain relief due to risks of open surgery. Reports have emerged describing minimally invasive variants to nearly every open thoracic approach to corpectomy [3, 11–15]. We present here the treatment options described in the literature, with an emphasis on specific advantages, disadvantages, and surgical nuance (Table 1).

## 2. Transthoracic

 Thoracotomy to access the anterior thoracic spine was first described in the 1950s [16]. Used initially primarily in the treatment of thoracic disc herniation, it found significant popularity in the 1970s and 1980s in response to the disappointing results for laminectomy for decompression and discectomy, due to poor outcomes associated with manipulation of the thoracic spinal cord [1, 6, 17–19]. Surgery involves placing patients in the lateral position, making a lengthy incision laterally along the associated rib, performing thoracotomy, and retracting the lung anteriorly. The parietal pleura is then split close to the rib head, allowing visualization of the costovertebral joint. The costovertebral ligaments and rib head are removed creating anterolateral visualization of the vertebral body, allowing discectomy and corpectomy. Closure includes leaving a chest tube, typically for three days of recumbent drainage [1, 17, 18]. While early reports showed good associated outcomes, surgical morbidity quickly prompted surgeons to explore other approaches [2, 5]. Approach related complications include pulmonary contusion, atelectasis, pleural effusion, chylothorax, and hemothorax [5, 7].

 Video-assisted thoracoscopy has allowed surgeons to avoid much of the incision- and dissection-related morbidity associated with thoracotomy [11, 20, 21]. Similar to thoracotomy, the patient is intubated with a double endotracheal tube with deflation of the ipsilateral lung, in a lateral position. Four thoracoscopic ports are placed via 2-centimeter incisions over the intercostal space, spaced widely throughout the chest, centered over the level of interest. The thoracoscope is typically a 10 mm fixed endoscope, with angled options available. Because the working distance to the spine ranges from 14 to 30 mm, specific adaptations of common surgical instruments are required, including drills, soft tissue dissectors, hemostatic agents, and spinal tools. Similarly to open thoracotomy, the appropriate costovertebral joint is identified, with subsequent opening of the pleura, removal of the rib head, discectomy, corpectomy, and reconstruction [11, 22] Closure consists of copious irrigation, inspection of the ipsilateral lung, followed by placement of chest tubes [11, 23–25].

 Yanni et al. recently described a variation of this approach, focused on alleviating the challenge of manually holding the endoscope [26]. They conducted a similar exposure with port placement, but once the exposure was complete, they utilized one of the ports to place a tubular retractor against the spine, under direct visualization with the endoscope. This allowed them direct lateral exposure comparable to the technique commonly used in the lumbar spine.

 The advent of thoracoscopy has allowed spine surgeons to reconsider the anterolateral approach to the thoracic spine [21]. Existing series suggest that the technique is feasible, and it appears to be as successful as open surgery in allowing decompression and instrumentation [21, 23]. Anterior visualization allows surgeons to perform complete corpectomy, visualizing the posterior longitudinal ligament, the entire anterior spinal cord, ipsilateral pedicle and foramen. The exposure allows a wide variety of grafts to be inserted, with the benefit of screw-plate fixation. Above T11, the surgeon can choose a right- or left-sided approach based on specific patient anatomy, to concentrate on visualization of affected critical structures including the azygos vein, aorta, thoracic duct, and artery of Adamkiewicz. T11 and T12 should be approached from the left to avoid the liver, and require caudal retraction of the hemidiaphragm [1, 11].

 Significant limitations persist, however, in the utilization of thoracoscopy. A steep learning curve has been described for surgeons beginning to undertake the technique [11, 27]. Intraoperative utilization of the multiple ports along with the endoscope can be facilitated by the use of fixed table based systems, but often can require significant assistant support. Introduction of a tubular retraction system may overcome this challenge, however [11, 26]. Working in a ventral-to-dorsal direction limits visualization of the posterior longitudinal ligament and thecal sac, and forces the surgeon to continuously estimate the distance between the working instrument and the spinal cord [3]. Additionally, patients with significant lung pathology limiting single lung ventilation or pleural adhesions are contraindicated from thoracoscopy [11].

 Despite the disadvantages, thoracoscopy has been shown to reduce the incidence of pulmonary morbidity, intercostal neuralgia, and shoulder girdle dysfunction versus open thoracotomy [8, 23, 28]. Patients suffer significantly less pain and incisional morbidity in thoracoscopic cases, with a lower rate of postthoracotomy pain syndrome [21]. Overall complication rates have been quoted to be significantly lower than those reported for thoracotomy, which ranges from 9 to 11.5% incidence of major complication [5, 7]. Nevertheless, the rate of complications including atelectasis, pneumothorax, hemothorax, and pleural effusion are still considerable, ranging from 14.1 to 29.4% [11, 29, 30]. Additionally, the burden of chest tube placement can still cause significant pain and limitation of postoperative patient mobilization.

## 3. Retropleural

 McCormick and Moskovitch described the retropleural approach to the anterolateral thoracic spine in the early 1990s as a method to avoid the morbidity associated with thoracotomy [31, 32]. Employing a retropleural approach allows for a ventral decompression without requiring entrance into the pleural cavity. McCormick's report described 15 patients undergoing treatment ranging from discectomy to two-level corpectomy. In his surgical technique, a 12 cm incision is performed from the posterior axillary line to 4 cm lateral of midline, with exposure and removal of 8–10 cm of the rib. The endothoracic fascia is incised and dissected off of the parietal pleura, leaving a plane with only slight areolar tissue, which is dissected until the endothoracic fascia is opened over the rib head. The costovertebral ligaments and proximal rib head are taken down to expose the vertebral body, facilitating corpectomy and reconstruction. Pleural tears are repaired primarily, and a chest tube is not required unless a significant tear is encountered. In the series of fifteen patients, adequate decompression and reconstruction were performed in all cases, although four patients did require chest tube placement.

 The significant exposure-related morbidity of this approach has limited its appeal and usage. Recent descriptions of a minimally invasive retropleural approach, however, have reopened the anterolateral corridor for corpectomy. Scheufler described a minimally invasive variant of the retropleural approach in 38 patients [33]. He made a 5-6 cm incision laterally, removed an 8–10 cm segment of the rib, and dissected between the endothoracic fascia and pleura towards the rib head. He then placed retracting blades in a 360-degree fashion and performed anterolateral corpectomy. Four out of thirty-eight patients ultimately required chest tube drainage, and all patients had adequate decompression and insertion of instrumentation.

 Uribe et al. furthered this approach by describing a tubular retractor based retropleural approach in a cadaveric series and a small patient series [12]. By using tubular dilators to perform the retropleural dissection, their series most closely adheres to MIS principals. They perform a 6 cm incision, remove 5 cm of underlying rib, and dissect free the retropleural plane towards the ribhead. Sequential tubular dilators are inserted, finishing with an expandable table-based retractor. Corpectomy is performed in a pedicle-to-pedicle fashion, with an anterior shell of bone and the ALL (anterior longitudinal ligament) preserved to protect thoracic contents. Reconstruction is performed with an expandable cage and autograft, with ventrolateral screw-plate fixation. A midline posterior incision is then performed, and posterior percutaneous screws are placed for reinforcement. One of their four reported cases required chest tube placement, and there were no perioperative complications. Kasliwal and Deutsch also described a similar approach for thoracic discectomy, utilizing a 2 cm incision to place an expandable tubular retraction system through a retropleural corridor in 7 patients [34]. A case report from Keshavarzi et al. also utilized this approach [35].

 Advantages of this approach include excellent anterior column reconstruction and little risk to the spinal cord. However, a significant challenge, particularly at the thoracolumbar junction, is manipulation of the diaphragm [36]. Dakwar and colleagues performed an anatomic study on 9 cadavers, examining the variants of diaphragmatic insertion points. They noted that while the diaphragmatic insertion is released with partial rib resection and mobilization of the pleura, by pursuing tubular dilation, the fibers of the diaphragm are not being cut. Thus, there is no need for repair of the diaphragm during closure [36]. However, other challenges associated with the retropleural approach include risk to the lumbar plexus, the mechanical difficulty of decompressing the canal from this angle, and risk to the segmental arteries.

## 4. Posterolateral

The lateral extracavitary approach was first described by Capener in 1954, and modified by Larson et al. [37, 38]. It has since been modified and popularized by numerous modern spine surgeons [39–42]. It provides a posterolateral, oblique approach to the vertebral body and spinal canal without entering the pleural cavity or retropleural dissection. The common description of the procedure describes a hockey stick incision, with the short limb extending 8 cm laterally of midline, in either prone or 3/4 prone position. The thoracodorsal fascia is exposed and the erector spinae muscles are elevated off the ribs. The rib is dissected free, cut 6–10 cm laterally, and removed after disarticulation of the costovertebral joint. With minimal retraction, discectomy and corpectomy can be performed under direct visualization, although the contralateral edge of the vertebral body and pedicle are not visualized. A wide variety of grafts can be introduced, and standard posterior pedicle screw/rod fixation achieved. A chest tube is only required in case of pleural violation [38–44].

**Figure 1:**
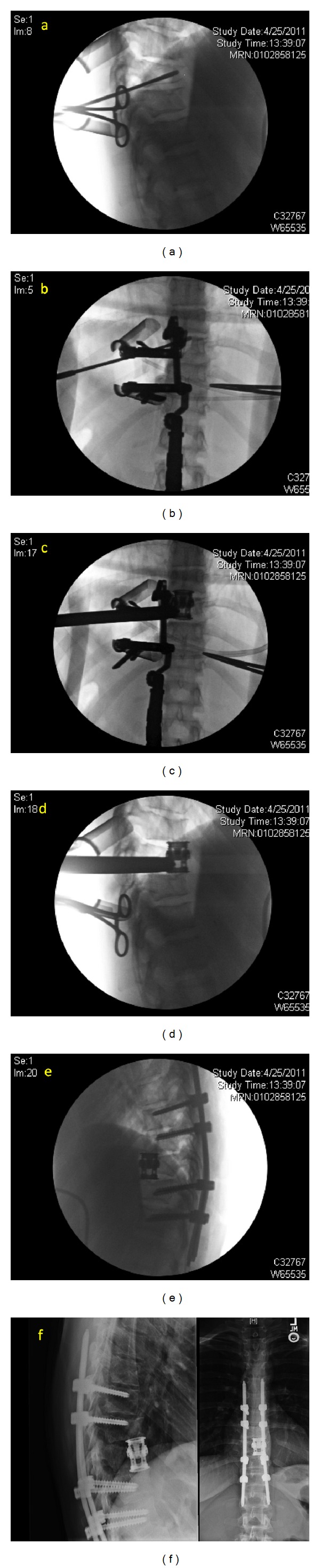
24-year-old female who suffered a traumatic T9 fracture and underwent MIS lateral extracavitary T9 corpectomy with T7-T11 posterior segmental instrumentation—Sequential intraoperative and postoperative images ((a)–(f)).

**Figure 2:**
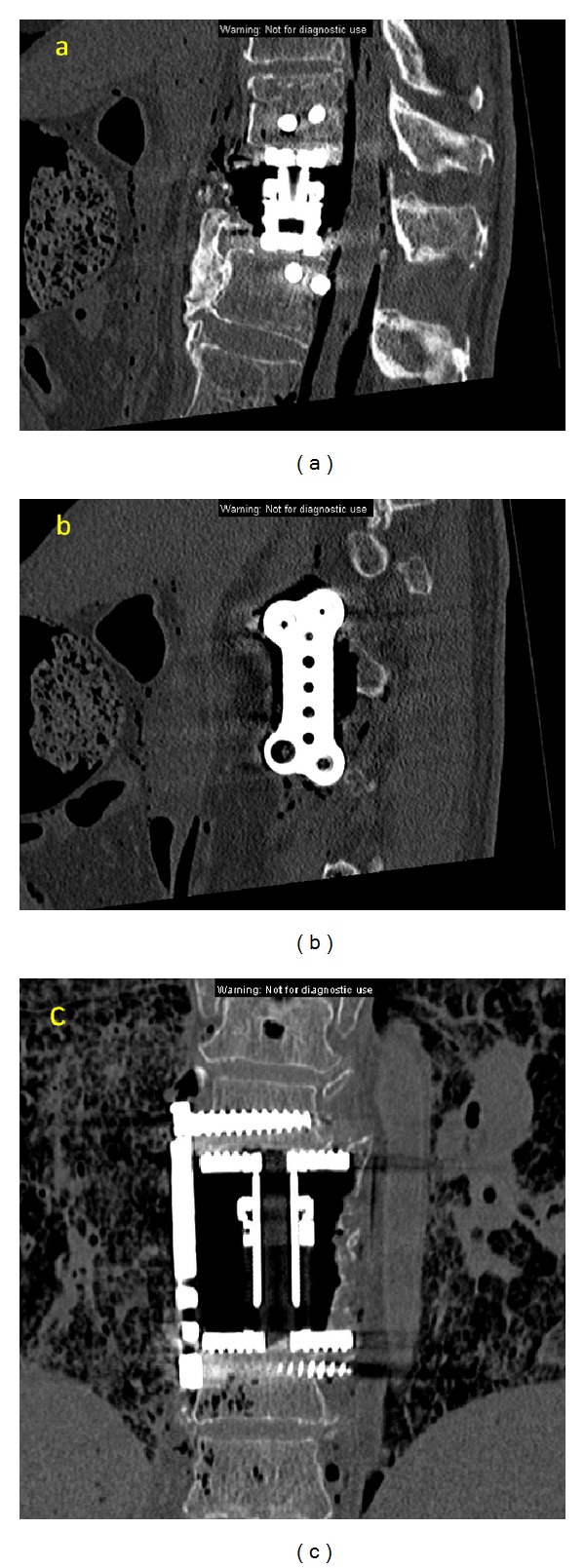
Cadaveric MIS lateral extracavitary corpectomy-coronal (a), and sagittal images of the plate (b) and cage (c) construct.

 Given the extensive muscle dissection required for the lateral extracavitary approach, the application of MIS techniques may provide distinct advantages. The first description of minimally invasive posterolateral corpectomy was published by Kim et al. in a series of cadaveric procedures and a small series of patients [3]. They started with a four cm long incision four cm laterally from midline. A K wire (Kirschner wire) was docked on the ipsilateral facet near the pedicle, followed by dilators and an expandable tubular retractor. The proximal rib was removed, followed by removal of the costovertebral ligaments, rib head, intercostal vessels, and ipsilateral pedicle. Discectomy and corpectomy were performed with preservation of the ventral body, ALL, and contralateral vertebral margins. Titanium mesh, autograft, or expandable cages were used for reconstruction, and vertebral body screws and rods were sometimes used for supplementation. Posterior percutaneous screws and rods were typically placed through a second incision. An average of 79.2% corpectomy was performed in the cadaveric cases, although the contralateral side was unable to be decompressed. Average estimated blood loss was 495 mL, operating room time was 5.8 hours, and hospital stay was 4.7 days in the clinical series. Satisfactory neural decompression was accomplished in all cases. Images from a representative case and cadaveric surgery are shown here (Figures 1 and 2).

 Khoo et al. described their experience with removal of thoracic disks and interbody fusion utilizing a similar minimally invasive posterolateral thoracic approach in 13 patients, and compared the cohort to patients undergoing traditional transthoracic surgery [45]. They utilized a 2 cm incision and docked the K wire at the junction of the ipsilateral transverse process and pedicle. They performed diskectomy without thecal sac retraction by rotating the bed in an oblique fashion. Their one-year outcomes were equivalent to open surgery, and most patient metrics favored the minimally invasive approach. Smith et al. described their outcomes in minimally invasive posterolateral corpectomy in a recent manuscript outlining a cadaveric series and surgical management of three patients. They utilized similar technique to Kim et al. They had appropriate decompression and instrumentation in all three patients, estimated blood loss of 517 mL, and average operating room time of 4.75 hours. They were able to achieve a mean of 72% vertebral body resection in the cadaveric series, without contralateral decompression [46]. Mussachio et al. published a cadaveric study, describing another variant of the approach [13]. They introduced a first tube to perform a costotransversectomy and corpectomy on the more affected side. They then placed a contralateral tube to perform contralateral transpedicular decompression. This technique allowed circumferential decompression by pursuing contralateral transpedicular completion of the corpectomy [13].

 The lateral extracavitary approach is one of the most widely validated approaches for corpectomy in the thoracic spine. Decompression and neurologic outcomes are excellent, and complications are typically minor and self-limited [39–41]. Nevertheless, muscle-dissection-related morbidity is severe, and the substantial tissue dissection and blood loss place severe systemic stress on the patient. One series described an average of 3100 mL of blood loss and 7.74 hours per case, although these numbers may have been exaggerated by a small number of complicated cases [39]. In contrast, minimally invasive posterolateral corpectomy appears to provide adequate decompression and instrumentation, with less blood loss and operative time [3]. An important advantage of this approach as opposed to midline posterior approaches is preservation of the midline posterior tension band. It also allows the ability to create longer constructs by placing percutaneous screws above and below the level of corpectomy. Nevertheless, the learning curve and patient morbidities may limit general applicability.

## 5. Posterior

 The transpedicular approach has been extensively utilized in patients whose comorbidities limit transthoracic and lateral extracavitary approaches [47–51]. Outcomes appear favorable when compared with other open techniques, and the technique has been described for a wide range of pathology [47, 50, 52–54]. Surgery consists of midline incision two levels above and below the level of pathology, with dissection to the lateral edge of the transverse processes. The posterior elements are removed, along with the bilateral facets, demonstrating the thecal sac and pedicles. The pedicles are then taken down, exposing the vertebral body for corpectomy and adjacent level discectomy. Multiple techniques have been described for placement of an expandable cages in the transpedicular approach: including thecal sac mobilization, rib head osteotomy, rib head disarticulation, and trap-door rib head osteotomy, with thinning of the rib to allow greenstick fracture and displacement with subsequent displacement [47, 52, 53].

 Deutsch et al. performed minimally invasive transpedicular corpectomy in 8 patients with metastatic tumors [14]. They focused on patients older than 68 years of age, who were deemed to be poor candidates for open surgery, with less than one year of life expectancy, but significant neurologic deficit. They first performed a 3 cm incision above the transverse process of the more affected side. They used sequential dilators and an expandable tubular retractor to visualize the posterior elements, then took down the ipsilateral transverse process, proximal lamina, and pedicle. They were able to decompress 75% of the canal from a single side. Bilateral decompression was performed when necessary to decompress the entire anterior canal. They did not instrument as they did not feel that stability had been compromised, and given the palliative nature of the procedures [14].

 Chou and Lu described minimally invasive transpedicular corpectomy with expandable cage reconstruction [15]. They describe the procedure for 8 patients and compare it to a similar open cohort. They perform a midline incision two levels above and below the level of interest, preserving the fascia. Percutaneous screws are placed two levels above and below the level of corpectomy. A midline fascial opening is performed over the level of interest, and an expandable tubular retraction system is placed. The posterior elements are removed, followed by removal of the pedicles and adjacent level diskectomy. They then perform bilateral transpedicular corpectomy. They perform a trap door rib head osteotomy, allowing expandable cage placement. They comment that removing the tubular retractor and placing a cerebellar helps to insert the cage, along with rotating the cage while inserting it between the vertebral bodies. They did not perform arthrodesis in these cases. Compared to their open cohort, they showed lower blood loss, similar operative time, and similar complication rates [15].

## 6. Discussion

 The varying approach corridors to the thoracic spine offer different advantages and drawbacks (Figure 5). The anterior (transthoracic and thoracoscopic) approaches allows the broadest decompression of the vertebral body with the ability to visualize the entire anterior thecal sac, but presents complications associated with entering the thorax, and risks related to working adjacent to the aorta and azygos vein [5, 23, 29, 55]. Working in a ventral-to-dorsal direction forces the surgeon to constantly estimate his distance to the thecal sac [3]. Learning thoracoscopy also demands specialized training from the surgeon [11]. The retropleural approach offers a similar view to thoracoscopy without entering the pleura, but even the existing minimally invasive descriptions require at least a 6–8 cm incision, substantial rib resection, and an extensive retropleural dissection [12, 33]. This dissection is technically demanding, results in an increased risk of pleural violation and chest tube placement, and may be mechanically more awkward than the transthoracic approaches [31].

The posterolateral approach allows surgeons to use a more familiar surgical angle (Figure 4). The minimally invasive variant spares much of the muscle dissection classically associated with the lateral extracavitary approach, decreasing blood loss, and surgical time [3, 45]. The lateral angulation allows the surgeon to directly visualize the thecal sac during decompression. Nevertheless, the unilateral approach likely limits the surgeon to a maximum of 80% corpectomy, and the contralateral pedicle, PLL, and ventral thecal sac cannot be clearly visualized in cadaveric studies [3]. Also, placement of percutaneous screws is typically required for reinforcement, which requires a second, parallel incision. This technique may also require a significant learning curve for the surgeon [3, 45]. The midline transpedicular approaches use a familiar midline trajectory, with either a miniopen approach through midline fascial opening, or bilateral expandable tubular retractors [13–15]. This approach allows bilateral decompression, cage reconstruction, and posterior instrumentation through a single exposure. Nevertheless, placement of the cage still requires either significant manipulation of the rib head or thecal sac, and working with the spinal cord directly between the surgeon and the vertebral body poses clear risks for injury [15, 49]. Loss of the midline posterior tension band may also result depending on the approach.

Choice of surgical approach carries implications regarding instrumentation implementation. Anterior and anterolateral approaches will dictate anterior only instrumentation systems, while posterolateral and posterior approaches better allow for posterior pedicle screws in the same position, with or without anterior cage reconstruction. Anterior approaches allow plating for stabilization over a wide variety of grafts, ranging from autograft to cages [11, 21]. The posterolateral approach allows for multiple types of anterior grafts as well, but supporting plate/screw systems are limited to a unilateral lateral orientation. As a result, most surgeons are performing a second incision for placement of percutaneous screws [3, 46]. The midline posterior approach is secured with percutaneous screws, with or without expandable cage grafting. In the posterior approach, supporting plating cannot be performed over the graft [15]. Studies have demonstrated that anterior-only constructs for thoracic reconstruction are feasible and appear at least as efficacious as posterior only constructs, although they may be less biomechanically sound [56, 57]. Anterior reconstruction has also been suggested to carry the advantage of correcting kyphosis and preventing secondary kyphosis [11, 57, 58].

 Descriptions of minimally invasive techniques for corpectomy are currently very limited by small sample size and limited followup. While some of the series have made early attempts to compare outcomes to the more established open procedures, comparisons are made only on the basis of intraoperative data such as blood loss and feasibility of decompression and instrumentation. Long-term instrumentation outcomes, fusion rates, and patient morbidity and mortality data are still lacking at this time. Thus, continued followup will be required before these minimally invasive techniques can be held in equipoise with established open procedures. Nevertheless, surgeons should be aware of these technical possibilities, and should consider their incorporation in modern surgical practice.

## 7. Lumbar and Thoracolumbar Corpectomy

**Figure 3:**
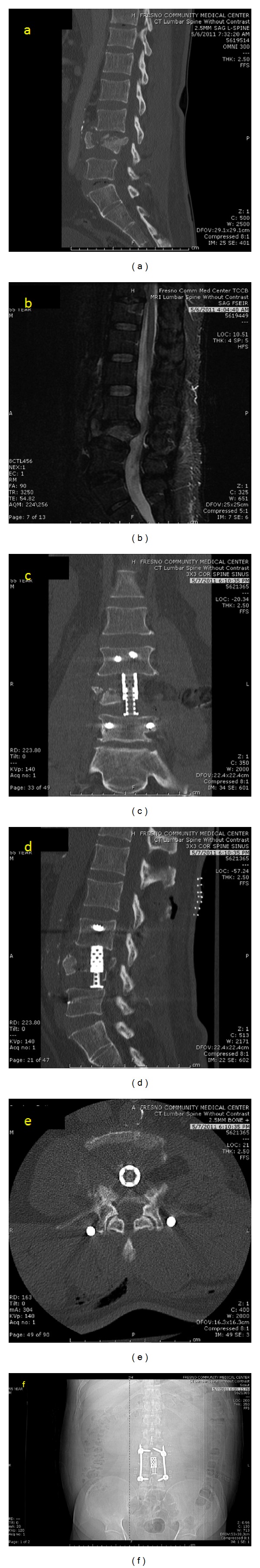
21-year-old who suffered a roll-over MVC and L4 burst fracture, and who underwent MIS lateral corpectomy: significant preoperative and postoperative images ((a)–(f)).

**Figure 4:**
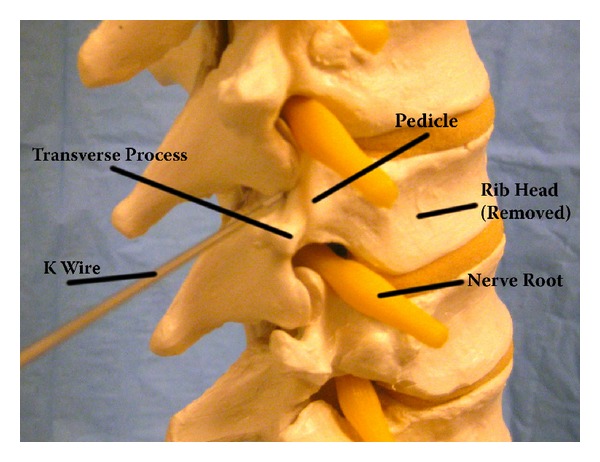
Saw bones image with a K wire showing the localization point for MIS lateral extracavitary corpectomy. Relevant anatomy highlighted.

**Figure 5:**
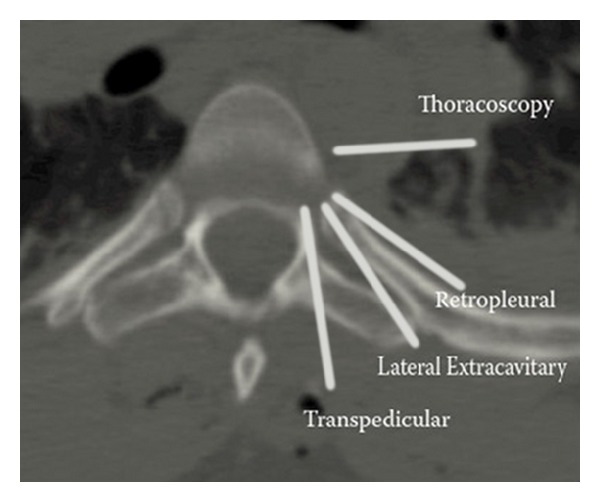
Axial CT image in midthoracic spine demonstrating the trajectory used in the various minimally invasive approaches for corpectomy.

 Comprehensive discussion of emerging techniques in lumbar and thoracolumbar corpectomy would easily command an independent paper. Nevertheless, most emerging techniques in minimally invasive lumbar corpectomy utilize similar principals to the thoracic techniques, specifically blunt tubular muscle and plane splitting, to minimize blood loss and tissue trauma. Lateral and anterior techniques in the lumbar spine and thoracolumbar junction provide similar advantages for decompression and reconstruction. We present an illustrative case of a 21-year-old male who suffered an L4 burst fracture and underwent MIS lateral corpectomy and reconstruction (Figure 3).

## 8. Conclusion

 Minimally invasive approaches for corpectomy in the thoracic spine offer substantial exposure-related advantages compared to their open counterparts. Descriptions are new and will require larger series and greater long-term followup to become fully validated. Choice of exposure approach should be driven by a patient's specific pathology, anatomy, and medical comorbidities.

## Figures and Tables

**Table 1:** Advantages and limitations of various minimally invasive approaches.

MIS approach	Selected authors	Advantages	Limitations
Anterior (thoracoscopic)	Dickman et al.	Complete decompression of canal	Pleural entry/chest tube
	Mack et al.	Easy graft insertion	Ventral to dorsal working pattern
	Ragel et al.	Anterolateral screw-plate fixation	High complication rates

Anterolateral (retropleural)	Uribe et al.	Complete decompression of canal	Extensive retropleural dissection
	Scheufler et al.	Anterolateral screw-plate fixation	Difficult working angle
	Kasliwal et al.	Extra-coelomic working corridor	High rate of pleural violation

Posterolateral (lateral extracavitary)	Kim et al.	Clear visualization of thecal sac	Significant blood loss/OR time
	Khoo et al.	Anterior stabilization	Unilateral decompression
	Mussachio et al.	Preservation of posterior tension band	Second incision for percutaneous stabilization

Posterior (transpedicular)	Chou et al.	Single incision	Difficult to place interbody graft
	Deutsch et al.	Circumferential decompression	Thecal sac between surgeon and body
		Decreased blood loss/pain	Dorsal to ventral working pattern (aorta, etc.)
